# Evaluating and refining the wheelchair mobility activity log (WC-MAL): a comprehensive study of validity and reliability

**DOI:** 10.1038/s41393-026-01170-9

**Published:** 2026-02-03

**Authors:** Tainara Rodrigues dos Santos, Jocemar Ilha, Carolina Luiza Donzelini Rodrigues, Aline de Lima, Thais Raquel Filippo, Natalia Duarte Pereira

**Affiliations:** 1 Research group on Functionality and technological innovation in neurorehabilitation (GFIT-Neuro), Department of Physiotherapy, Universidade Federal de São Carlos, São Carlos, SP Brazil; 2 Spinal Cord Injury Rehabilitation Research Group (SCIR-Group), Department of Physiotherapy, Universidade do Estado de Santa Catarina (UDESC), Florianópolis, Santa Catarina, Brazil

**Keywords:** Rehabilitation, Spinal cord diseases

## Abstract

**Study design:**

Validity and Reliability Analysis.

**Objective:**

Evaluate the structural validity, reliability and criterion validity of the Wheelchair Mobility Activity Log (WC-MAL) using Rasch analysis.

**Methods:**

Sixty individuals with SCI and using a manual wheelchair participated in the study. The WC-MAL was employed remotely. Rasch analysis evaluated the structural validity of the instrument. The intra-rater reliability of the WC-MAL score was analysed using the random model Intraclass Correlation Coefficient (ICC) and the Standard Error of Measurement was calculated to estimate the precision of individual scores. For concurrent criterion validity, the data from the tachometer were used as the “gold standard” to assess wheelchair mobility, with the WC-MAL serving as the comparator. Pearson’s correlation coefficient was used to evaluate the relationship between the tachometer data and WC-MAL Frequency Scale scores.

**Results:**

The Rasch analysis led to the exclusion of three items (1, 3, and 10) from the original instrument, improving model fit and refining WC-MAL 2.0. WC-MAL 2.0 demonstrated good discriminant ability with a Person Separation Reliability of 0.91–0.93 and explained variance between 59.3 and 61%. The WC-MAL 2.0 showed no local dependency, maintained unidimensionality across all scales, and exhibited no uniform Differential Item Functioning. The WC-MAL 2.0 demonstrated excellent inter-rater reliability (ICC0.84–0.91), strong internal consistency (Cronbach’s alpha 0.84–0.91), and strong correlations between the Frequency Scale and tachometer data (r = 0.78, p < 0.001), supporting its criterion validity.

**Conclusion:**

The WC-MAL 2.0 is a suitable instrument with adequate validity and reliability for assessing wheelchair performance in individuals with SCI.

## Introduction

Managing the mobility limitations caused by Spinal Cord Injury (SCI) often requires the use of a wheelchair [1, 2], as less than half of individuals with SCI achieve independent walking [3]. It is crucial to explore a wide range of skills and ensure the effective use of a wheelchair [4]. Mastering various manual wheelchair skills is essential for independence in daily activities, autonomy in challenging environments, social participation, and safe community mobility, all of which contribute to an improved quality of life [5–7].

The literature offers various instruments for assessing manual wheelchair mobility and skills [8]. Among these, the Wheelchair Mobility Activity Log (WC-MAL) stands out as a semi-structured interview specifically designed to evaluate wheelchair use during mobility activities in real-world settings. The WC-MAL instrument and its development process have been previously documented and validated in individuals with SCI [9] and are available in “www.udesc.br/cefid/nuleme/wcmal”. It targets individuals with SCI who rely on a manual wheelchair as their primary means of mobility. The WC-MAL includes items that cover daily wheelchair mobility activities, using standardized questions to assess the frequency, performance, and assistance needed for each activity in the individual’s actual environment (i.e., the real-world). This means that the WC-MAL measures the self-reported actual use of the wheelchair over the past week, capturing the individual’s “lived experience” in their usual context [9].

Distinctive features of the WC-MAL include its emphasis on real-world mobility activities, the integration of items aligned with the International Classification of Functioning, Disability and Health (ICF), and adherence to the COnsensus-based Standards for the selection of health Measurement Instruments (COSMIN) guidelines for the development of self-report instruments [9–11]. Additionally, the WC-MAL showed adequate content validity evaluated through the evaluation of relevance, comprehensiveness, and comprehensibility by individuals with SCI and rehabilitation professionals during instrument development.

Although content validity is an essential measurement property of a developed instrument [12], other properties must be evaluated before the instrument can be fully recommended for use and the results obtained with it can be trusted, especially the internal structural validity [12]. The COSMIN guidelines [13] emphasize several key aspects of the internal structural validation of measurement instruments using Rasch analysis [14–16]. First, they recommend ensuring unidimensionality, meaning that all items on a scale measure a single construct. The guidelines suggest that each item should fit the Rasch model, with poorly fitting items potentially needing revision or removal. Differential Item Functioning (DIF) is another critical aspect, for which the guidelines advocate for assessing whether different respondent groups (e.g., based on age or gender) interpret and respond to items similarly, to avoid bias. The hierarchical ordering of items based on difficulty provided by Rasch analysis should also be theoretically and clinically meaningful [14–16]. Furthermore, COSMIN highlights the importance of construct validity, demonstrating that items measure the intended theoretical construct [12, 17].

While Rasch analysis goes beyond traditional internal consistency measures like Cronbach’s alpha, the COSMIN still stresses the importance of evaluating internal consistency, other validation properties and inter-rater reliability [13]. These recommendations ensure that the instruments are robust, reliable, and valid, providing precise and useful measures in clinical practice and research [13]. Thus, this article aims to evaluate the structural validity, internal consistency, intra-rater reliability, and concurrent criterion validity of the WC-MAL for assessing mobility activities among individuals with SCI.

## Methods

### Study design

This is a descriptive, quantitative study with a cross-sectional design conducted to assess the internal structural validity of the WC-MAL using Rasch analysis, a probabilistic model grounded in item response theory. Additionally, the reliability and criterion validity were assessed. This study was conducted according to the Declaration of Helsinki and approved by the Ethical Committee of the Universidade Federal de São Carlos (3.710.548). All participants provided informed consent.

### Participants and data collection

This study used a non-probabilistic convenience sample. Convenience sampling was chosen because the instrument is specifically designed for the assessment of individuals with SCI. Participants were recruited through social media, radio advertisements, and project flyers. Inclusion criteria were adults with SCI, chronic injury duration (>6 months), and use of a wheelchair as the primary means for daily mobility. Exclusion criteria included not being enrolled in any specific wheelchair skills or mobility training program.

A sample size of 60 was targeted for the internal structural validity analysis to ensure sufficient statistical power for Rasch analysis. This sample was deemed appropriate based on the general assumption that the minimum sample size for polytomous observations is at least 50 participants. For Rasch modelling, a sample size of 60 provides useful and stable estimations of item and person locations irrespective of scale targeting. [18, 19] This sample size allows adequate testing of dimensionality of the instrument and ensures that the model can accurately evaluate the relationships between items and the underlying construct.

For data collection, two independent physiotherapists (TRS and CLD, raters #1 and #2 respectively) administered the WC-MAL via video calls, with sessions conducted two weeks apart. This time was deemed adequate once the participants were considered clinically stable and were not enrolled in any specific training to improve their mobility in wheelchairs.

The raters read the manual of WC-MAL and were trained for its application by the senior author. They were blinded to each other’s scores to ensure unbiased evaluations. The data from rater#1 were used for the internal structural validity (Rash analysis) and intra-rater reliability through concordance analysis with the data from rater #2.

Following the second WC-MAL interview, a home visit was arranged to install a tachometer on the wheelchair wheel in those individuals who were living in São Carlos City (SP, Brazil), enabling the measurement of movement and wheelchair use over three days.

### Wheelchair mobility activity log (WC-MAL)

The WC-MAL is a semi-structured interview that comprises 23 activities (items) assessed by three scales: the Frequency Scale, Performance Scale, and Assistance Scale. Respondents are asked about their wheelchair use for each activity listed in the instrument. If the respondent answers “yes” to having performed the activity within the past week, the activity is then scored using the three scales. Each scale ranges from 0 to 5, with 0 indicating non-performance and 5 indicating the highest frequency, best performance, and greatest independence for the activity, respectively. If the response is “no,” the respondent is asked to provide a reason, which can be selected from a predefined list of general possible causes.

At the conclusion of the interview, individual scores are obtained for each of the three scales, and a composite score is calculated by averaging the scores from each scale. Each of the three WC-MAL scales ranges from 0–5, with higher scores indicating greater frequency, better performance, or greater independence. The composite score, calculated as the mean of the three scale scores, also ranges from 0 to 5, reflecting an overall measure of mobility across the assessed activities [9].

### Rasch analysis

The Rasch measurement model was employed to assess the structural validity of the WC-MAL. For this analysis, the Winsteps program (Version 3.92.1, Winsteps, Beaverton, Oregon, USA) was used, focusing on the Frequency Scale, Performance Scale, and Assistance Scale scores. This model estimates a person’s ability relative to item difficulty, expressed in log-odds units (logits) on a single continuum scale. Participants with higher ability and more difficult items are positioned on the same end negative side of the continuum scale, and vice versa. Item fit statistics are expressed using infit and outfit mean square (MNSQ) statistics, based on the chi-square statistic with each observation weighted by its statistical information (model variance). A range of 0.7–2 is used as a criterion for good fit.

Targeting refers to how well the difficulty of items matches the abilities of the study sample. The standard error of the person measures was used for assessment. Cutoff points were defined as follows: fair targeting (1–2 error), good targeting (<1 error), and very good targeting (<0.5 error). Differential Item Functioning (DIF) analysis, including both uniform and non-uniform DIF, was performed to identify significant differences in item responses by subgroups based on demographic characteristics. DIF was assessed by gender, type of lesion (complete or incomplete), level of lesion (cervical, thoracic, and lumbar), and presence or absence of shoulder pain. Non-uniform DIF indicates unstable behavior in item response probability due to an external factor, suggesting potentially biased responses. Notable DIF was defined as a difference of >1.0 logits [20, 21].

Local dependency was identified through paired standardized residual correlations between items exceeding 0.30 [21]. If local dependency occurs, it is recommended to combine the dependent items into one. Measurement precision was assessed by person separation reliability (PSR) and the separation index (PSI). Person separation is used to classify individuals, and low person separation in a relevant person sample implies that the instrument may not be sensitive enough to distinguish between higher and lower performers. A PSR of ≥0.80 (PSI ≥ 2.00) indicates that the instrument can distinguish the study population into two to three levels of disability [21].

The unidimensionality of the Rasch model is assessed by independent t-tests for each person, with less than 5% of tests outside the ±1.96 range indicating a unidimensional scale. In principal components analysis of the residuals (PCA), 60% of the variance explained by the raw data is considered evidence of unidimensionality. An eigenvalue in the first contrast of the residuals >2.0 suggests the need to measure a second construct [22].

### Reliability and criterion validity

The intra-rater reliability of the composite WC-MAL score was analyzed using the random model Intraclass Correlation Coefficient (ICC [1,1]) with 95% confidence intervals (95% CI), considering the following levels of agreement: weak (ICC < 0.40); moderate (ICC = 0.40- 0.75) and excellent (ICC > 0.75) [23]. Additionally, the Standard Error of Measurement (SEM) was calculated to provide an estimate of the precision of individual scores in the assessment.

The internal consistency of the WC-MAL was evaluated using Cronbach’s alpha coefficient, a commonly used measure to assess the reliability of scales. Cronbach’s alpha quantifies the extent to which the items within a scale are interrelated, indicating the instrument’s internal reliability. An alpha value of 0.70 or higher was considered acceptable.

For the concurrent criterion validity analysis, the data from the tachometer and the WC-MAL Frenquency Scale scores were compared. The concurrent criterion validity was evaluated using Pearson’s correlation coefficient (r) between the tachometer data and the WC-MAL Frequency Scale scores. Correlation magnitudes were classified as moderate to good (r = 0.50–0.75) or strong (r ≥ 0.75) [23]. A strong positive correlation was hypothesized to exist between both assessments.

## Results

### Participants

The sample, comprising 60 individuals diagnosed with SCI (paraplegia or tetraplegia), of both sexes, aged ≥ 16 years, and using a manual wheelchair for mobility, participated in the study to evaluate the structural validity of the WC-MAL through Rasch analysis and inter-rater reliability (Table 1). Additionally, 33 individuals with a tachometer installed on their wheelchair wheel comprised the sample for criterion validity.

**Table 1:** Characteristics of Participants: 60 Individuals in the Structural Validaty Study and Reliability and 33 Individuals in the Criterion Validity Study.

	n = 60	Mean ± s.d.	n = 33	Mean ± s.d.
Sex				
Men	40 (66, 7%)		24 (75%)	
Women	20 (33, 3%)		8 (25%)	
Age (year)		41,6 ± 9,3		44,10 + /− 8,01
Chronicity (year)		11,8 ± 8,1		12,14 + /− 8,37
Level of injury				
Cervical	22 (36,7%)		13 (40,62%)	
Thoracic	32 (53,3%)		17 (53,12%)	
Lumbar	6 (10,0%)		2 (6,25%)	
Type SCI				
Complete	31 (51,6%)		20 (62,5%)	
Incomplete	29 (48,3%)		12 (37,5%)	
Injury mechanism				
Traumatic	53 (88, 3%)		29 (90,62%)	
Non-traumatic	7 (11, 7%)		3 (9,37%)	
Physical Activity				
Men	29 (72,5%)		16 (89%)	
Women	7 (35%)		2 (11%)	

### Rasch analysis

After performing the Rasch analysis, items that did not fit the model for the three evaluation scales were excluded. Subsequently, a new Rasch analysis was conducted with the remaining items to develop a revised version, the WC-MAL 2.0. The original and revised version (WC-MAL 2.0) are organized in Table 2 according to the hierarchy of item difficulty for Frequency Scale, Performance Scale, and Assistance Scale, from easiest to most challenging items. The corresponding initial and revised Rasch analysis values, excluding the non-fitting items, are presented in Table 3.

**Table 2:** Goodness-of-fit statistics for the 23- and 20-item versions of the WC-MAL. Items with 0.7>MnSq>1.3 (underscored data) present a problematic fit to the Rasch model.

No.	Item	Frequency					Performance					Assistance				
		Infit		Outfit		Model S.E.	Infit		Outfit		Model S.E.	Infit		Outfit		Model S.E.
		MnSq	Z	MnSq	Z		MnSq	Z	MnSq	Z		MnSq	Z	MnSq	Z	
**WC-MAL**																
19	Move indoors	0.92	−0.1	0.83	−0.3	1.3	0.66	−1.0	0.63	−1.0	2.21	0.93	0.0	0.67	−0.4	1.5
2	Transfer to and from surfaces of the similar height	0.73	−0.6	0.7	−0.7	1.4	0.67	−1.2	0.65	−1.1	2.14	0.74	−3.8	0.70	−2.3	0.8
4	Transfer to the bath or shower	1.26	−1.2	0.89	−1.4	2.1	0.72	−1.8	0.63	−1.8	1.21	0.72	−4.9	0.76	−2.5	0.91
11	Reach for objects in front of you at shoulder height	0.77	−1.7	0.79	−1.8	0.7	0.55	−1.7	0.61	−1.2	1.29	0.81	−0.2	1.16	0.5	1.10
14	Open and go through doorways	0.98	0	0.82	−0.5	2.0	0.70	−1.2	0.78	−0.6	0.93	0.66	−1.0	0.72	−0.6	0.93
13	Open and close doors	1.11	0.5	0.86	−0.4	1.5	0.92	−0.2	0.65	−1.1	1.70	1.14	0.5	0.51	−0.9	0.65
6	Transfer to and from the car	0.86	−0.5	0.71	−1	1.8	0.76	−1.7	0.59	−1.6	1.50	0.37	−4.5	0.71	−2.2	1.30
**1**	Remain seated for long periods*	**1.94**	**3.4**	**2.61**	**4.3**	**1.1**	**1.50**	**2.0**	**1.87**	**2.4**	**1.05**	**1.87**	**2.6**	**2.17**	**2.0**	**0.89**
8	Pick up small objects from the floor	1.07	0.4	0.97	0	0.98	0.81	−0.9	0.72	−1.0	0.93	0.85	−0.6	0.59	−1.0	0.95
18	Navigate narrow places	1.08	0.5	0.89	−0.4	1.4	1.25	1.1	1.17	0.6	1.35	1.54	1.9	1.05	0.3	1.20
12	Reach for objects above shoulder height	0.69	−1.8	0.78	−2.1	0.79	0.79	−1.1	0.65	−1.5	1.47	0.90	−0.5	0.85	−0.3	0.67
15	Go up and down sloped surfaces	0.58	−2.7	0.77	−2.2	0.50	0.68	−1.9	0.79	−0.8	1.37	0.77	−2.1	0.59	−1.5	0.42
7	Transport large objects while pushing the wheelchair	1	0.1	0.99	0	1.9	1.10	0.6	1.25	1.1	1.48	1.47	2.1	1.09	1.9	0.38
20	Move through busy environments	0.76	−1.6	0.72	−1.4	1.7	1.18	1.0	1.14	0.6	1.07	1.08	0.5	0.97	0.0	0.97
16	Go up and down a step	0.92	−0.5	0.8	−0.9	1.1	0.97	−0.1	0.89	−0.4	0.93	0.81	−1.1	0.84	−0.5	1.14
23	Use transportation as a passanger	1.22	2.5	1.52	2.1	1.1	1.26	3.0	1.14	3.6	1.24	1.30	1.7	1.21	1.7	1.42
21	Navigate uneven surfaces	0.9	−0.6	0.81	−0.8	0.5	0.78	−1.3	1.01	0.2	0.92	0.67	−2.1	0.71	−1.4	1.5
**10**	Carry a large item on the back of the wheelchair*	**1.41**	**2.4**	**1.91**	**3.2**	**1.2**	**1.51**	**2.8**	**1.83**	**2.9**	**0.99**	**1.52**	**2.9**	**1.53**	**1.8**	**0.99**
**3**	Transfer to and from the toilet*	**1.59**	**2.6**	**1.62**	**1.8**	**0.2**	**1.77**	**3.3**	**1.90**	**2.1**	**0.92**	**1.77**	**3.0**	**1.91**	**2.2**	**0.92**
22	Travel long distances	1.26	1	1.34	0.9	0.9	1.16	0.8	1.08	0.3	1.01	1.13	0.7	1.07	0.3	0.89
9	Pick up large objects from the floor	1.28	1	1.3	0.8	1.3	1.60	2.1	1.09	1.9	1.2	1.24	1.9	1.65	1.5	0.88
5	Transfer to and from the floor/ground	0.96	0	0.93	0	0.2	0.83	−0.6	0.70	−0.5	0.7	1.01	0.1	0.90	−0.1	0.57
17	Go up and down a flight of stairs	0.94	0	0.76	−0.2	1.3	1.21	0.7	0.79	−0.1	1.2	1.39	1.0	0.85	0.0	0.46
**WC-MAL 2.0**																
19	Move indoors	0.96	0.0	0.99	0.1	1.3	0.96	−1.0	0.93	−1.0	2.21	0.83	0.0	0.77	−0.4	1.5
2	Transfer to and from surfaces of the similar height	0.78	−0.5	0.87	−0.2	1.4	0.77	−1.2	0.85	−1.1	2.14	0.74	−**3.8**	0.70	−**2.3**	0.8
11	Reach for objects in front of you at shoulder height	0.68	−1.2	0.57	−1.5	0.7	0.72	−1.8	0.83	−1.8	1.29	0.72	−**4.9**	0.76	−**2.5**	1.10
4	Transfer to the bath or shower	0.71	−1.1	0.67	−1.1	2.1	0.85	−1.7	0.91	−1.2	1.21	0.91	−0.2	1.16	0.5	0.91
13	Open and close doors	1.22	0.9	0.91	−0.2	1.5	0.70	−1.2	0.78	−0.6	1.70	0.66	−1.0	0.72	−0.6	0.65
14	Open and go through doorways	1.02	0.2	0.84	−0.4	2.0	0.92	−0.2	0.85	−1.1	0.93	1.14	0.5	0.91	−0.9	0.93
6	Transfer to and from the car	0.95	−0.1	0.79	−0.7	1.8	0.76	−1.7	0.59	−1.6	1.50	0.57	−**4.5**	0.71	−**2.2**	1.30
8	Pick up small objects from the floor	1.23	1.1	1.13	0.6	0.98	0.81	−0.9	0.92	−1.0	0.93	0.85	−0.6	0.89	−1.0	0.89
18	Navigate narrow places	1.32	1.5	1.06	0.3	1.4	1.25	1.1	1.17	0.6	1.35	**1.54**	1.8	1.25	0.3	0.95
12	Reach for objects above shoulder height	0.80	−1.1	0.66	−1.6	0.79	0.79	−1.1	0.85	−1.5	1.47	0.90	−0.5	0.85	−0.3	1.20
15	Go up and down sloped surfaces	0.73	−1.5	0.74	−1.1	0.50	0.68	−1.9	0.89	−0.8	1.37	0.77	−**2.1**	0.99	−1.5	0.67
7	Transport large objects while pushing the wheelchair	1.17	1.0	1.17	0.8	1.9	1.10	0.6	1.25	1.1	1.48	0.99	**2.1**	1.09	1.9	0.42
20	Move through busy environments	0.84	−0.9	0.81	−0.9	1.7	1.08	1.0	1.14	0.6	1.07	1.08	0.5	0.97	0.0	0.38
16	Go up and down a step	1.03	0.2	0.91	−0.3	1.1	0.97	−0.1	0.89	−0.4	0.93	0.81	−1.1	0.84	−0.5	0.97
23	Use transportation as a passanger	1.22	**2.5**	**1.52**	**2.1**	1.1	1.26	**3.0**	1.14	**3.6**	1.24	1.30	1.7	1.21	1.7	1.14
21	Navigate uneven surfaces	1.09	0.6	1.03	0.2	0.5	1.26	**3.0**	1.04	**3.6**	0.92	1.30	1.7	1.21	1.7	1.42
22	Travel long distances	1.12	1.6	1.27	1.3	0.9	0.78	−1.3	1.01	0.2	1.01	0.67	**−2.1**	0.81	−1.4	0.89
9	Pick up large objects from the floor	1.33	1.2	**1.82**	1.6	1.3	1.16	0.8	1.08	0.3	1.2	1.23	0.7	1.07	0.3	0.88
5	Transfer to and from the floor/ground	0.92	−0.2	0.92	0.0	0.2	**1.60**	**2.1**	1.09	1.9	0.7	1.24	1.9	1.25	1.5	0.57
17	Go up and down a flight of stairs	0.96	0.0	0.61	−0.5	1.3	0.83	−0.6	0.90	−0.5	1.2	1.01	0.1	0.90	−0.1	0.46

The WC-MAL 2.0 was obtained after eliminating problematic items. The bold numbers indicate statistical values outside the adequate reference ranges. The asterisks (*) indicate the eliminated WC-MAL problematic items.

**Table 3:** Summary statistics for Rasch analyses of the original WC-MAL and the revised WC-MAL 2.0.

Scales	# of itens	Rasch model fit		Targeting (SE of person measure)	Measurement precision		Local dependence	Unidimensionality		
		Infit MNSQ Mean (Range)	Outfit MNSQ Mean (Range)		PSR	PSI		% Significant t-test	% Variance explained by measures	Unexplained variance in 1st contrast (Eigenvalue)
**Frequency**										
WC-MAL	23	1.20 (0.8 – 1.9)	1.00 (0.3 – 1.4)	0.88	0.89	6.91	−0.78	0.05	61%	1.6
WC-MAL2.0 Remove items 1, 3 and 10	20	1.04 (0.89 – 1.24)	1.01 (0.72 – 1.03)	0.85	0.93	7.14	−0.98	0.05	60.8%	1.52
**Performance**										
WC-MAL	23	0.88 (0.3 – 1.1)	0.95 (0.4 – 1.8)	0.92	0.88	6.52	−0.82	0.06	58.8%	1.7
WC-MAL2.0 Remove items 1, 3 and 10	20	1.02 (0.92 – 1.19)	1.10 (0.85 – 1.20)	0.93	0.91	6.58	−0.98	0.05	59.3%	1.8
**Assistance**										
WC-MAL	23	1.00 (0.5 – 1.2)	1.20 (0.5 – 1.6)	0.89	0.88	5.96	−0.88	0.05	61.1%	1.2
WC-MAL2.0 Remove items 1, 3 and 10	20	1.05 (0.90 – 1.15)	1.03 (0.89 – 1.15)	0.85	0.91	6.26	−0.99	0.05	61%	1.3
**Ideal Values**		**(0.70 – 1.30)**	**(0.70 – 1.30)**	**<1 error**	**≥0.80**	**≥ 2.0**	**No positive correlation** > **0.30**	**≤0.05**	**>60.0**	**<2.00**

*The items 1, 3 and 10 were removed with infit/outfit MNSQ indicating misfit the Rasch model.

Analysis of the original WC-MAL items showed that the infit/outfit MNSQ values for items 1 (“Sitting for long periods”), 3 (“Transferring to and from the toilet”), and 10 (“Transporting a large volume behind the wheelchair”) were outside the acceptable MNSQ range 0.7–1.3. Removal of items 1, 3 and 10 significantly improved the model fit compared with the initial model, as indicated by the likelihood ratio test (Table 3).

The modified WC-MAL, after removal of these items, is now referred to as WC-MAL 2.0 and exhibited a PSR of 0.91–0.93 and PSI of 6.26–7.14, suggesting strong discriminant ability of the questionnaire. The instrument WC-MAL 2.0 demonstrated adequate targeting, although the residuals for the three scales explained 59.3–61% of variance, which is slightly lower than the recommended value. The raw variance and 0.05 of the significant t-tests indicated unidimensionality of the Frequency Scale, Performance Scale, and Assistance Scale. The unexplained variance in first contrast ranged from 1.3 to 1.8 eigenvalue units, indicating no evidence of another latent trait captured by the scale. No local dependency was detected with all paired standardized residual correlations <0.30 (Table 3).

All items of the WC-MAL were free from uniform DIF. However, notable non-uniform DIF was detected in items 1 (“Sitting for long periods”) and 23 (“Using transportation as a passenger”). For item 1, men demonstrated greater execution ability (1.2 logits), while individuals with shoulder pain showed more difficulty (1.3 logits). For item 23, individuals with lumbar spinal injuries exhibited higher execution ability (1.2 logits).

### Reliability and criterion validity

The WC-MAL 2.0 scales showed excellent inter-rater reliability (ICC, 0.84–0.91) and internal consistency (Cronbach’s alpha, 0.84–0.91). The SEM ranged from 0.25 to 0.34 (Table 4).

**Table 4:** Inter-rater reliability and internal consistency of the WC-MAL 2.0 frequency, performance, and assistance scales.

Scales	Mean (SD) Rater 1	Mean (SD) Rater 2	ICC (IC 95%)	Alfa de Combrach	SEM
Frequency	2.75 (0.77)	3.07 (0.64)	0.85 (0.69 - 0,93)	0.85	0.30
Performance	2.82 (0.84)	3.16 (0.82)	0.84 (0.67 – 0.92)	0.84	0.34
Assistance	2.98 (0.85)	3.17 (0.84)	0.91 (0.82 – 0.96)	0.91	0.25

Standard error of measurement (SEM) for the frequency, performance, and assistance scales.

Additionally, the Frequency Scale score of WC-MAL 2.0 (mean 2.4; s.d. 1.2), showed strong correlations with the number of rotations recorded by the tachometer (mean 1309.9; s.d. 513.1) over three consecutive days (r = 0.78, p < 0.001).

## Discussion

The WC-MAL 2.0 demonstrated adequate structural validity. The study confirmed the unidimensionality of the WC-MAL 2.0 subscales through Rasch model fit tests. Results showed unique information from most items, satisfactory correlation between overall individual capacity and task performance scores, and effective applicability of the item test with articulated task categories. The subscales effectively differentiated task difficulty levels and individuals with varying functional levels, aligning with a theoretically expected hierarchical order of tasks. The study supported the hypothesis that WC-MAL 2.0 fits the Rasch model for individuals with SCI who are manual wheelchair users. Additionally, WC-MAL 2.0 showed excellent criterion validity, inter-rater reliability, and internal consistency.

The analysis identified and hierarchically ordered items from easier to more difficult tasks, revealing that task difficulty influenced activity performance in individuals with bodily limitations. Other studies have also found that advanced skills are more challenging and less frequently performed by participants due to the level of difficulty [24, 25]. In our results, participants with cervical injury levels were aligned with the easiest instrument activities and had greater difficulty in performing more complex items. In a previous study [26], most volunteers with tetraplegia were unsuccessful in more challenging skills, such as climbing a 15 cm curb and ascending a 10° slope, and only 28.6% of participants with tetraplegia demonstrated good performance in the wheelchair mobility activities that required advanced wheelchair skills. In our study, climbing stairs was the most challenging activity, consistent with other studies reporting the difficulty of advanced wheelchair skills. Participants with cervical injuries faced greater difficulty with complex tasks, confirming the complexity and motor demands of advanced skills.

Fliess-Douer et al. and Coolen et al. have categorised wheelchair skills into levels of difficulty [27, 28], including basic and daily living skills such as reaching, self-propulsion on flat and short distances, simple manoeuvres like anterior and posterior displacement, turning with the wheelchair, parking, etc. In our study, the easiest activities included moving through indoor environments and reaching forward at shoulder height, aligning with basic wheelchair skills required for these activities. The DIF analysis revealed that personal characteristics like sex, injury type, and shoulder pain had little influence on item responses.

The COSMIN group established the criteria for good measurement properties for each domain. For the instrument to demonstrate sufficient measurement properties, all criteria must be met. If any criteria are not met the measurement property is considered insufficient, and if any criteria do not report it is  considered indeterminate. For structural validity analyzed by Rasch, the instrument must not violate unidimensionality, local independence and monotonicity, and must demonstrate adequate model fit [29]. Our results did not indicate violations of these criteria and therefore support the sufficient structural validity of WC-MAL 2.0.

The criteria for internal consistency require at least low-quality evidence for sufficient unidimensionality and Cronbach’s alpha values greater than 0.70 for each subscale [29]. Our results demonstrated sufficient unidimensionality, and all subscales showed good Cronbach’s alpha values, indicating sufficient internal consistency.

Additionally, the WC-MAL 2.0 showed excellent inter-rater reliability. The criterion for reliability is an ICC greater than 0.70 [29] indicating sufficient reliability. For measurement error, classification depends on minimal important change (MIC) [29]. Our study did not aim to analyze interpretability, so we recommend that future studies analyze the MIC to be able to compare the values with our findings and classify the measurement error. However, it is noteworthy that the measurement error was less than seven per cent of the WC-MAL 2.0 scale values (from 0.25 to 0.34 of 5.00).

The criterion validity of the WC-MAL 2.0 was supported, as the Frequency Scale scores of WC-MAL were strongly correlated with the number of rotations recorded by the tachometer over the recorded time. The criteria for good measurement properties determined a correlation with a gold standard greater than 0.70, [29] which indicates a sufficient criterion validity.

Furthermore, for an instrument to achieve a high level of clinical recommendation, all relevant measurement properties must be sufficient with high-quality evidence. [29] A systematic review has shown that no current instrument has evidence for all measurement properties, particularly for content validity and internal structural validity [8]. WC-MAL 2.0 has evidence of content validity, and we demonstrated high-quality evidence for sufficient internal structural validity, reliability and criterion validity. So, the WC-MAL 2.0 can be considered the clinical instrument with the greatest current potential for assessing the wheelchair mobility performance among individuals with SCI in both clinical and research settings. The measurement property analysis that is still missing for the full WC-MAL 2.0 recommendation is responsiveness, and cross-cultural and hypotheses testing validities. So future studies must address these missing properties to create high-quality evidence of WC-MAL 2.0 measurement properties and allow the high-rated recommendation for its use.

The WC-MAL 2.0 instrument proved  suitable for assessing mobility in manual wheelchair users with SCI, demonstrating good reliability and validity. The revised version, organised hierarchically, is practical for both research and clinical use without requiring rigorous training. Future studies should consider measuring physical activity levels, assess in-person application feasibility and evaluate additional measurement properties.

## Strengths and limitations

We applied robust statistical analysis to prove sufficient measurement properties of the WC-MAL with methodological rigour. The results can be generalised for manual wheelchair users with SCI. Studies need to be conducted with a representative sample of wheelchair users with other health conditions, due to differences in population characteristics that influence wheelchair use.

We recognize that the WC-MAL includes a limited number of items directly related to active wheelchair propulsion, while most items address functional tasks involving upper limb use, postural control, and overall wheelchair management. This restricts the extent to which distance-based measures can represent true functional mobility. Therefore, the concurrent criterion validity assessed in this study should be interpreted as preliminary, and more comprehensive evaluations could incorporate functional activity instruments, such as PROMIS or SCI Model Systems measures.

## Conclusion

In conclusion, the WC-MAL 2.0 is a reliable and valid instrument for assessing mobility in manual wheelchair users with SCI, providing valuable insights into the impact of personal characteristics on wheelchair mobility in the real-world.

## Data Availability

The data will be made available by the corresponding author upon reasonable request.
